# Prevention of human rabies: a challenge for the European Union and the European Economic Area

**DOI:** 10.2807/1560-7917.ES.2020.25.38.2000158

**Published:** 2020-09-24

**Authors:** Céline M Gossner, Alexandra Mailles, Inma Aznar, Elina Dimina, Juan E Echevarría, Siri Laura Feruglio, Heidi Lange, Francesco Paolo Maraglino, Patrizia Parodi, Jurijs Perevoscikovs, Yves Van der Stede, Tamás Bakonyi

**Affiliations:** 1 European Centre for Disease Prevention and Control (ECDC), Stockholm, Sweden; 2 Santé publique France, Saint Maurice, France; 3 European Food Safety Authority (EFSA), Parma, Italy; 4 Centre for Disease Prevention and Control of Latvia, Riga, Latvia; 5 Centro Nacional de Microbiología, Instituto de Salud Carlos III, Madrid, Spain; 6 Centro de Investigación Biomédica en Red de Epidemiología y Salud Pública (CIBERESP), Madrid, Spain; 7 Norwegian Institute of Public Health, Oslo, Norway; 8 Ministry of Health, Rome, Italy

**Keywords:** rabies, vaccination, dog, bats, travel, lyssaviruses

## Abstract

Rabies is enzootic in over one hundred countries worldwide. In the European Union/European Economic Area (EU/EEA), the vast majority of human rabies cases are travellers bitten by dogs in rabies-enzootic countries, mostly in Asia and Africa. Thus, EU/EEA travellers visiting rabies enzootic countries should be aware of the risk of being infected with the rabies virus when having physical contact with mammals. They should consider pre-exposure vaccination following criteria recommended by the World Health Organization and if unvaccinated, immediately seek medical attention in case of bites or scratches from mammals. As the majority of the EU/EEA countries are free from rabies in mammals, elimination of the disease (no enzootic circulation of the virus and low number of imported cases) has been achieved by 2020. However, illegal import of potentially infected animals, mainly dogs, poses a risk to public health and might threaten the elimination goal. Additionally, newly recognised bat lyssaviruses represent a potential emerging threat as the rabies vaccine may not confer protective immunity. To support preparedness activities in EU/EEA countries, guidance for the assessment and the management of the public health risk related to rabies but also other lyssaviruses, should be developed.

## Background

Rabies is a viral zoonosis that is enzootic in over one hundred countries around the world [1]. Rabies causes around 59,000 human deaths annually; 95% of those occur in Asia and Africa. There is a wide spectrum of clinical symptoms, ranging from furious to paralytic manifestations. Without prophylaxis, the fatality rate is near 100% [1].

Rabies lyssavirus (RABV) is present in the saliva of infected animals up to 10 days before onset of symptoms and is transmitted through contact between the saliva and wounds (e.g. following a bite or scratch) or mucosal surfaces (e.g. licking of mucosa) [1,2]. In Asia and Africa, human deaths result almost entirely from transmission of the virus by dogs; in the Americas, human deaths are mostly caused by bat-associated RABV [1]. Human-to-human transmission through donation of contaminated organs and tissues has occurred [3].

There are safe and effective human vaccines that are used both as pre- and post-exposure prophylaxis. The World Health Organization (WHO) recommends pre-exposure prophylaxis (PrEP) for (i) people who are at high risk of exposure to RABV and other lyssaviruses because of their profession or their engagement in particular activities (e.g. animal healthcare workers; people doing caving) and, (ii) people travelling to remote areas where timely access to adequate post-exposure prophylaxis (PEP) cannot be guaranteed or if the individual is at high risk of contact with wild animals, particularly bats [1]. The type of contact with the suspected lyssavirus-shedding animal determines the indicated PEP procedures which consist of thorough washing and flushing of the wound, prompt initiation of a post-exposure vaccination scheme, and, if indicated, administration of immunoglobulins. With a prompt and proper PEP, exposed patients have a survival rate close to 100% [1].

This perspective article on human rabies in the European Union/European Economic Area (EU/EEA) aims to (i) provide an overview of the current epidemiological situation in humans and animals in the EU/EEA; (ii) highlight the risk among travellers globally and within the region (iii) present prevention opportunities and challenges for countries; and (iv) suggest actions for surveillance and preparedness. While the main focus is on rabies, we briefly address the risk related to other lyssaviruses.

## Human rabies cases

Based on data collected by the European Centre for Disease Prevention and Control and a non-systematic review, we concluded that there have been, 18 travel-related cases of rabies in the EU/EEA between 2006 and 2019; ranging from none (e.g. in 2015) to four (in 2019) cases per year (Table 1). Detailed information on the data search is provided in the supplementary material. The mean age was 44 (interquartile range: 38–55). The female-to-male ratio was 0.6:1. All cases were infected in rabies-enzootic countries. The mean incubation period was around 5 months. All cases died of their infection.

**Table 1:** Characteristics of the travel-related rabies cases reported in Europe, 2006–2019 (n = 18)

Year of symptom onset	Number of human cases	Country of residence	Country of infection	Region^a^ of infection	Prompt post-exposure prophylaxis	Animal exposure	Estimated incubation period	References
2006	1	Lithuania	India	Southern Asia	No	Dog	Unknown	[4]
2007	2	Germany	Morocco	Northern Africa	No	Dog	ca 6 weeks	[4,41]
		Finland	Philippines	South-eastern Asia	No	Dog	< 2 months	[4,42]
2008	1	United Kingdom	South Africa	Sub-Saharan Africa	No	Dog	24 months	[4,43]
2011	2	Portugal	Guinea Bissau	Western Africa	No	Dog	< 3 months	[4,44]
		Italy	India	Southern Asia	Yes^b^	Dog	< 1 month	[24]
2012	1	United Kingdom	India	Southern Asia	No	Dog	< 3 months	[4,45]
2013	1	The Netherlands	Haiti	Caribbean	No	Dog	< 2 months	[4,46]
2014	3	The Netherlands	India	Southern Asia	Yes^b^	Dog	ca 8 weeks	[4,47]
		France	Mali	Western Africa	No	Unknown	1 to 6 months	[4,48]
		Spain	Morocco	Northern Africa	No	Dog	6 months	[4,49]
2016	1	France	Bangladesh	Southern Asia	No	Dog	Unknown	[4]
2017	1	France	Sri Lanka	Southern Asia	No	Dog	< 2 months	[4,50]
2018	1	United Kingdom	Morocco	Northern Africa	No	Cat	2 months	[4,51]
2019	4	Norway	Philippines	South-eastern Asia	No	Dog	< 2 months	[4,52]
		Latvia	India	Southern Asia	No	Dog	18 months	[4,52]
		Spain	Morocco	Northern Africa	No	Cat	4 months	[4,52]
		Italy	Tanzania	Eastern Africa	Yes^b^	Dog	1 month	[4,52]

^a^ Based on the United Nations Statistics Division [45].

^b^ Vaccination only, in the country of exposure.

Countries that reported cases: Finland (n = 1), France (n = 3), Germany (n = 1), Italy (n = 2), Latvia (n = 1), Lithuania (n = 1), the Netherlands (n = 2), Norway (n = 1), Portugal (n = 1), Spain (n = 2) and the United Kingdom (n = 3).

Seven of the travel-related cases were infected in southern Asia (i.e. India, Bangladesh, Sri Lanka), four in northern Africa (i.e. Morocco), two in western Africa (i.e. Mali, Guinea Bissau), two in south-eastern Asia (i.e. Philippines), one in eastern Africa (i.e. Tanzania), one in Sub-Saharan Africa (i.e. South Africa), and one in the Caribbean (i.e. Haiti) . Sixteen cases were infected through dog bites and two cases through cat bites. To our knowledge, none of the cases had received PrEP. Fifteen cases did not receive prompt PEP after exposure and three cases received incomplete PEP in the country of infection (i.e. no immunoglobulins were provided).

In addition, during the studied period, France and Romania reported locally acquired (non travel-related) rabies cases. The case in France resided in the overseas department of French Guiana where he got infected in 2008 [4]. In Romania, there was on average one case per year until 2012, the year of the last locally acquired infection in the country [4].

## Animal rabies cases

### Rabies in terrestrial mammals

The most important reservoir host of RABV in Europe is the red fox (*Vulpes vulpes*) [5]. Rabies in dogs was progressively eliminated at the turn of the 20th century in most EU/EEA countries. As this large achievement in the domestic population took place, an epidemic in red foxes as a result of a spill over event from domestic animals to red foxes commenced in the 1940s [6]. Attempts to interrupt the virus spread by reducing the red fox population below a threshold (so that intraspecific transmission was halted) failed because the necessary threshold was not reached [7].

Since the end of 1980s and following a number of successful vaccine field trials, a large oral rabies vaccination campaign in red foxes commenced in the EU. This campaign has been politically supported and co-financed by the EU and EU countries and covers not only these countries but also some of the EU’s neighbouring countries along its eastern and south-eastern borders (Table 2) [8,9]. By the time this campaign was launched, the EEA countries Iceland and mainland Norway were rabies-free among red foxes. Vaccination in Liechtenstein was not co-financed but the country participated to a joint vaccination campaign with Switzerland and obtained the rabies free status in 1986 [10].

**Table 2:** Description of rabies national veterinary programmes in 12 European Union countries and number of animal cases, 2019 [8]

Country	Doses of vaccines	Vaccine type	Vaccine coverage area (km^2^)	Number of animal cases in 2019
Bulgaria	2,874,950	Lysvulpen (SAD, Bern, Switzerland)	23 regions: 57,019 km^2^	0
Estonia	280,600	Rabitec (strain SPBN GASGAS, IDT Biologika GmbH, Dessau-Rosslau, Germany)	Border facing Russia: 6,100 km^2^	0
Greece	2,980,200	Lysvulpen	Parts of the country: 59,604 km^2^	0
Finland	Finland: 180,000 Russia: 30,970	Rabitec	South-eastern border facing Russia: 10,000 km^2^	0
Croatia	2,665,850	Lysvulpen	Whole continental area without Adriatic islands: 53,317 km^2^	0
Hungary	Hungary: 1,680,000 Ukraine: 510,000	Rabigen SAG2 oral suspension (VIRBAC S.A.,Carros, France)	Hungary: 9 regions bordering Romania, Serbia, Croatia, Slovakia and Ukraine: 41,970 km^2^ Ukraine: 10,200 km^2^	0
Lithuania	Lithuania: 1,010,000 Belarus: 1,650,000	Lysvulpen	Border facing Russia and Belarus. In Lithuania: 20,400 km^2^ In Belarus: 33,000 km^2^	0
Latvia	Latvia: 962,250 Belarus:542,500	In Latvia Lysvulpen (Bioveta A.S., Ivanovice na Hané,Czech Republic); In Belarus Rabivac-O/333 (Pokrov biological plant, Volginsky, Russia)	Border facing Russia and Belarus. In Latvia: 19,245 km^2^ In Belarus: 10,850 km^2^	0
Poland	Poland: 6,007,728 Belarus: 604,000 Ukraine: 1,922,500	Lysvulpen; Rabigen SAG2 oral suspension; Rabitec	Poland: 8 regions 100,564 km^2^ Belarus: 12,080 km^2^ Ukraine: buffer zone with Poland 50km, 38,450km^2^	1 (red fox)
Romania	Romania: 10,819,550 Ukraine: 1,134,500 Moldova: 3,720	Romania: Lysvulpen Ukraine: Brovarabies V-RG (Ukrvetprompostach, Brovary, Ukraine) Moldova: Lysvulpen	Romania: whole country, 213,375 km^2^ Ukraine: 100 km from border: 22,500 km^2^ Moldova: 50 km from border, 14,560 km^2^	4 (2 red foxes, 1 wild boar and 1 cow)
Slovakia	632,800	Lysvulpen	Borders facing Hungary, Ukraine and Poland: 12,708 km^2^	0
Slovenia	760,000	Rabitec	Borders facing Croatia and Hungary: 7,800 km^2^	0

Oral rabies vaccination campaigns have been remarkably successful in controlling the infection in red foxes. In the EU/EEA in 2019, only a small number of animal cases occurred, all in eastern EU countries, and achieving elimination of rabies in mammals (no enzootic circulation of the virus and low number of imported cases) has been achieved by 2020 [11]. Between 2010 and 2019, the highest proportion of animal cases were found in red foxes (Table 3). A gradual decrease in the total number of cases among this species was observed over the years. Since 2017, the overall yearly number of rabies cases has been below 10 and only four EU countries have reported cases: Lithuania, Romania, Poland and Hungary [11]. In addition, Norway reported the detection of rabies cases among Arctic foxes on the islands of Svalbard and Jan Mayen.

**Table 3:** Number of animal rabies cases reported from EU/EEA countries in foxes, raccoon dogs, farmed animals, dogs and cats, 2010–2019 (n = 3,323)

Animals	2010	2011	2012	2013	2014	2015	2016	2017	2018	2019	Total
Foxes (*Vulpes vulpes and Vulpes lagopus*^a^)	643	331	503	544	319	99	14	2	6	3	2,464
Raccoon dogs (*Nyctereutes procyonoides*)	15	11	4	0	1	3	1	0	0	0	35
Farmed mammals^b^	75	51	70	85	56	11	10	3	1	1	363
Dogs (*Canis lupus familiaris*)	41	47	65	74	27	14	3	1	1	0	273
Cats (*Felis catus*)	42	29	45	39	18	12	2	1	0	0	188
Total	816	469	687	742	421	139	30	7	8	4	3,323

EU/EEA: European Union/European Economic Area.

^a^ Norway (Svalbard and Jan Mayen) reported five, two and four cases in Arctic foxes (*Vulpes lagopus*), in 2011, 2012 and 2018, respectively; all other cases were red foxes.

^b^ Farmed mammals: sheep, goats, pigs, solipeds, farmed pet animals other than dogs and cats.

Source: Data for 2010 to 2018 were reported to the European Food Safety Authority using the Data Collection Framework; data for 2019 were reported via the Animal Disease Notification System of the European Commission. Data downloaded on 17 December 2019.

Note: Other species found infected in small numbers are not included in the table (among others, badgers, deer, marten, rodents, jackals, lynx, bears, hares, hedgehogs, minks, wolverine, wild boar, squirrels, ferrets, otter, polecat, etc.).

In 2019, five animal rabies cases were reported, four in Romania (two in red foxes, one in a cow and a wild boar, respectively) and one in Poland (red fox) [12]. The case in Poland was found in proximity to the border with Belarus and Ukraine. In Romania, one of the red foxes was found close to the border with Hungary and Ukraine, and the other animal cases near the border with Moldova. In Ukraine in 2019, 260 cases of domestic rabies cases were reported, 101 in dogs and 138 in cats, and 262 cases were reported in wild animals, 232 of them were red foxes. No data were reported for Moldova or Belarus.

### Bat rabies

Rabies virus has not been found in native bats of Europe but has been found in other continents and is a major source of human infections in the Americas [13,14].

## Infections with other lyssaviruses

Lyssaviruses other than RABV have been detected in the EU/EEA and EU neighbouring countries. The European bat lyssavirus type 1 and 2 (EBLV-1 and EBLV-2, respectively) have bats as their main host and have been linked, in rare occasions, to infections in other animals and humans. In 2018, a total of 45 EBLV-1 and EBLV-2 bat cases were detected in France, Germany, Hungary, the Netherlands, Poland, Spain and the United Kingdom (UK) [15]. EBLV-1 has been detected in sheep in Denmark [16], in a stone marten in Germany [17], as well as in cats in France [18].

Four human deaths of EBLV-1 and EBLV-2 have been recognised so far in Europe: Ukraine (1977), Russia (1985), Finland (1985) and the UK (2002) [19].

To date, four other bat lyssavirus species were detected in the EU/EEA and their neighbouring countries, the Bokeloh bat lyssavirus, the Lleida bat lyssavirus*,* the West Caucasian bat lyssavirus and the Kotalahti bat lyssavirus (tentative species). No human cases were so far associated to these four other bat lyssaviruses [20]. In 2020, for the first time, a cat, who had a suspected exposure to bats, was tested positive for the West Caucasian bat lyssavirus in Italy [21].

In addition, one fatal human case of Duvenhage lyssavirus infection was diagnosed in the Netherlands in 2007 [22]. The person was bitten by a bat while she was in Tsavo West National Park, Kenya.

## Risk related to rabies

### Risk for travellers visiting rabies enzootic areas

For the majority of EU/EEA countries, rabies has become a disease of travellers being bitten or scratched by dogs or cats in countries with uncontrolled dog- and cat-derived rabies. Four travel-related human cases of rabies were reported in the EU/EEA in 2019. This is the highest number of cases reported in a year but only represents a slight increase compared to 2014 when there were three cases. This slight increase is not considered to reflect a change in the risk for travellers as there is no indication of a recent increase of the incidence of rabies in the reported countries of infection. However, we believe that the four cases reported in 2019 may highlight a lack of awareness among EU/EEA travellers, as it has been described by Marano et al. [23].

Based on reported data there are two groups of individuals potentially at higher risk of being exposed and/or contracting the disease: first, people who handle puppies and kittens and do not consider it a risk of exposure; second, people who are bitten/scratched by dogs or cats but do not seek medical attention.

In this regard, travel clinics and public health authorities in the EU/EEA may reinforce their prevention campaigns, advising travellers visiting countries with moderate and high risk of rabies (i) to be aware of the possibility of acquiring RABV infection when having physical contact with mammals, (ii) to get PrEP vaccination following criteria recommended by WHO and (iii) to immediately seek medical attention in case of bites or scratches from mammals. Dedicated communication campaigns should be developed for different groups of travellers and levels of awareness and the use of social media to reach them should be explored. In addition, travellers should be reminded to follow veterinary rules and regulations when travelling with pets. Furthermore EU/EEA citizens should only acquire pets through authorised channels.

### Pre- and post-exposure vaccination

To our knowledge, none of the travel-related cases reported in the EU/EEA had received PrEP and very few received prompt, but incomplete PEP after exposure. Several case reports highlighted that injured travellers who sought medical attention in countries considered at medium and high risk for rabies exposure did not receive adequate PEP, either because vaccines and/or immunoglobulins were unavailable or they were improperly administered [24-26]. Three of the travel-related cases reported in the EU/EEA had sought medical attention after exposure and received PEP in the country of exposure (i.e. India and Tanzania) but the scheme was incomplete and all developed rabies. Travellers from the EU/EEA receiving PEP in endemic countries should seek medical attention when returning to their country in order to check the adequacy of the treatment received.

Several studies have looked into the causes of non-vaccination of travellers. The cost of the vaccine, the lack of knowledge about the risk among travellers and healthcare providers and, the relatively long time to complete the vaccine course were the most frequent causes of being non-vaccinated [27]. Since 2018, the WHO recommends a vaccination schedule of 1 week, with only two doses, hence reducing the planning complexity and cost for travellers [28]. While the vaccine might still be considered expensive (up to EUR 100 per dose), the resulting immunity is long-lasting and the investment should be considered attractive for travellers who travel repetitively to rabies-enzootic areas [28]. In addition, PEP for vaccinated patients has been simplified: two vaccine doses over 3 days and no immunoglobulins are needed, an important point that healthcare providers in the EU/EEA should take into account considering that several EU/EEA countries have recently experienced temporary but occasionally recurrent shortages in vaccines and/or human immunoglobulins [29-31]. Although these shortages were resolved, national authorities had to temporarily restrict the use of the vaccine for PEP [32,33]. The provision of falsified vaccines has been highlighted as a public health issue as recently demonstrated outside the EU/EEA, e.g. in the Philippines and in China [34].

In 2018, the WHO and its partners have in their ‘zero-by-30’ initiative set the goal to end human deaths from classical rabies by 2030 [35]. Through a One Health approach, the initiative focuses on carnivores’ vaccination, control of stray dogs, access to PEP for exposed people and rabies education and awareness. However, for the initiative to be successful, there is an increasing need of affordable vaccines and immunoglobulins in countries considered at medium and high risk of RABV infection.

### Risk related to substances of human origin

Rabies virus is not found in blood; there is nonetheless a risk related to the transplantation of organs and tissues from infected donors in whom rabies was not diagnosed. Such events are fortunately very rare thanks to donor’s selection criteria implemented in most countries [36,37].

### Risk related to domestic carnivores

A considerable risk of human rabies still exists within the EU/EEA with the illegal importation of domestic carnivores (mainly dogs) from non-rabies free countries, especially northern Africa [38,39]. For instance, France identified in February 2020 a rabid dog illegally imported from Morocco [40].

### Risk related to other lyssaviruses

Bat lyssaviruses represent a potential emerging threat in the EU/EEA. Few fatal human cases of EBLV-1 and EBLV-2 have occurred in the EU, but the most concerning is the potential emergence of newly recognised bat lyssaviruses for which the pathogenicity for humans is unknown and for which the rabies vaccine may not confer protective immunity [20]. The recent detection of the West Caucasian bat lyssavirus in a cat with clinical symptoms in Italy indicates that emerging lyssaviruses may potentially infect other terrestrial mammal species including humans. Although this remains an isolated event, there is a need to promote further research including for the development of rabies vaccines covering a wide range of lyssaviruses.

Even though bats may be present on the roof or attic of buildings or homes in the EU/EEA, interactions between bats and humans are expected to be rare. As a general precaution, it is recommended to avoid contacts with bats and their excreta and seek medical attention in case of accidental exposure.

## Conclusion

As rabies among mammals is nearly eliminated from the EU/EEA, the risk for EU/EEA citizens is mostly related to travel to enzootic areas. Travellers should be aware of this risk, consider pre-exposure vaccination following recommended criteria by the WHO and those unvaccinated, should immediately seek medical attention in case of bites or scratches from mammals. Dedicated communication campaigns should be developed for travellers in risk groups. Even though exposures are generally limited, bat lyssavirus infection represents a potential emerging threat in the EU/EEA.

To support preparedness activities in EU/EEA countries, guidance for the assessment and the management of the public health risk related to rabies but also other lyssaviruses should be developed. Maintaining surveillance of animal and human rabies in countries or regions free from terrestrial rabies is essential to be able to early detect and efficiently manage any re-occurrence or re-emergence of the disease. Continuation of oral vaccination campaigns at the EU/EEA bordering regions with regular resurgence of rabies would reduce the risk of rabies re-emergence. This is particularly important at the eastern borders of the EU, as rabies is still enzootic in neighbouring countries.
